# Betulinic Acid Inhibits Growth of Cultured Vascular Smooth Muscle Cells *In Vitro* by Inducing G_1_ Arrest and Apoptosis

**DOI:** 10.1155/2012/251362

**Published:** 2012-09-27

**Authors:** Raja Kumar Vadivelu, Swee Keong Yeap, Abdul Manaf Ali, Muhajir Hamid, Noorjahan Banu Alitheen

**Affiliations:** ^1^Institute of Bioscience, Universiti Putra Malaysia, Selangor, Serdang, Malaysia; ^2^Faculty of Agriculture and Biotechnology, Universiti Sultan Zainal Abidin, Kampus Kota, Jalan Sultan Mahmud, 20400 Kuala Terengganu, Malaysia; ^3^Department of Microbiology, Faculty of Biotechnology and Biomolecular Sciences, Universiti Putra Malaysia, Selangor, 43400 Serdang, Malaysia; ^4^Department of Cell and Molecular Biology, Faculty of Biotechnology and Biomolecular Sciences, Universiti Putra Malaysia, Selangor, 43400 Serdang, Malaysia

## Abstract

Betulinic acid is a widely available plant-derived triterpene which is reported to possess selective cytotoxic activity against cancer cells of neuroectodermal origin and leukemia. However, the potential of betulinic acid as an antiproliferative and cytotoxic agent on vascular smooth muscle (VSMC) is still unclear. This study was carried out to demonstrate the antiproliferative and cytotoxic effect of betulinic acid on VSMCs using 3-[4,5-dimethylthizol-2-yl]-2,5-diphenyltetrazolium bromide (MTT) assay, flow cytometry cell cycle assay, BrdU proliferation assay, acridine orange/propidium iodide staining, and comet assay. Result from MTT and BrdU assays indicated that betulinic acid was able to inhibit the growth and proliferation of VSMCs in a dose-dependent manner with IC_50_ of 3.8 **μ**g/mL significantly (*P* < 0.05). Nevertheless, betulinic acid exhibited G_1_ cell cycle arrest in flow cytometry cell cycle profiling and low level of DNA damage against VSMC in acridine orange/propidium iodide and comet assay after 24 h of treatment. In conclusion, betulinic acid induced G_1_ cell cycle arrest and dose-dependent DNA damage on VSMC.

## 1. Introduction

Vascular smooth muscle cells (VSMCs) are the prime cellular component of the normal artery as well as of intimal lesions that develop in response to arterial injury. Consequently, proliferation and migration of VSMCs are hallmarks of vascular disorders such as atherosclerosis and restenosis [1]. Uncontrollable proliferation of VSMCs possessed similarity with tumor and benign tissue overgrowth. Recently, an improved outcome with using an attractive alternative to bare-metal stents is drug-eluting stent such as sirolimus-, rapamycin-, and paclitaxel-Eluting stents (TAXUS)-IV. These techniques demonstrated striking reductions in angiographic restenosis and revascularization rates with sirolimus-, rapamycin-, or paclitaxel-eluting stents, respectively [2]. However, comparative clinical trials have shown that drug-eluting stent does not confer any benefit in clinical outcomes [3] and may even predispose to stent thrombosis [4]. For example, higher concentration of paclitaxel may lead to increased apoptosis in the vessel wall and consequently to a more unstable phenotype of the preexisting atherosclerotic lesion [5]. On the other hand, sirolimus-eluting stents were not shown to effect on arterial pathology but it was described temporarily lead to systemic concentrations that approach immunosuppressive levels [6]. Thus, application of a nontoxic antiproliferative compound will be interesting to prevent restenosis. In the last decade, the implication of natural compound such as goniothalamin in controlling the proliferation and migration of neointima in diseased arthery has been widely studied [7].

Betulinic acid (BA) (3*β*-hydroxy-lup-20(29)-en-28-oic acid), is a pentacyclic lupane triterpene, isolated from various plant sources widespread throughout the tropics. It is a known natural compound shown to possess several medical properties [8]. Accumulated experimental evidences have revealed that BA has variety of biological activities including anti-inflammatory [9], anti-HIV, antibacterial [10], antimalarial [11], antiangiogenic [12], and antioxidant properties [13]. BA and its synthetic derivatives were shown to be a cytotoxic compound on variety of cancerous cell lines [14–19]. For example, BA was found as a cytostatic compound that induced the accumulation of B16F10 melanoma cells in the G_1_ phase [14]. Apoptosis induction initiated by BA was not exerted through a ligand/receptor system but it occurs through perturbation of mitochondrial function, such as loss of the mitochondrial transmembrane potential that decrease mitochondrial permeability [15] release of mitochondrial cytochrome c into the cytosol. Moreover, Fulda et al. [15] revealed that BA-induced apoptosis was found to be independent of p53 in melanoma and neuroectodermal tumor cells. Thus, BA directly altered mitochondria and induced loss of the mitochondrial transmembrane potential associated with inhibition of topoisomerases to provoke cell death of various cancerous cells [16]. Furthermore, betulinic acid was able to improve the effect of tumor radiotherapy under hypoxic condition [17].

Modulation of vascular smooth muscle (VSMCs) proliferation and migration has critical therapeutic implication for vascular disease. In order to find a potent inhibitor for VSMCs proliferation and migration, cytotoxicity of BA was tested in VSMCs cultures *in vitro*. Although our previous study has reported that BA was not toxic towards the normal cell human peripheral blood mononuclear cell and mice NIH-3T3 [18], BA has shown their potential to inhibit proliferation and migration of VSMC through inducing the expression of cyclins/CDKs and reducing the expression of p21(waf1/cip1)/p27(kip1), MMP-2, MMP-9 that finally downregulated ROS/NF-*κ*B signaling in a recent study [20]. Besides, Yoon et al. [21] also reported that BA could suppress TNF-*α*-induced vascular inflammatory process, due to the inhibition of ROS and NF-*κ*B activation in HUVEC. Thus, this study was aimed to correlate the cytotoxicity and genotoxicity contributed by the antiproliferative effect of BA on VSMCs.

## 2. Materials and Methods

### 2.1. Chemical and Reagents

Smooth muscle Basal Media (SmBM), insulin, hFGF-B, gentamicin, amphotericin-B, hEGF, and fetal bovine serum were purchased from Cambrex Bio Science Walkersville, Inc. (Walkersville, MD, USA). Na_2_HPO_4_ *·*7H_2_O, KH_2_PO_4_, Na_2_EDTA, DMSO were obtained from Ajax Laboratories and NaCl, KCl from British Drug House Limited. MTT, acridine orange, propidium iodide, trypan blue were purchased from Fisher Scientific. Mg^2+^- and Ca^2+^-free PBS tablet, normal melting agarose, (NMA), low melting agarose (LMA), ethidium bromide, Tris, and Triton X-100 were obtained from Sigma (USA). Betulinic acid extract was prepared by phytochemical laboratory of Universiti Putra Malaysia. Tissue culture dishes and flasks were purchased from Nunclon (Roskilde, Denmark). All other reagents and solvents were of analytical grade.

### 2.2. Cell Culture

Briefly, primary coronary artery smooth muscle cells (VSMCs) (ATCC cat. No.: PCS-100-021) were cultured under standard tissue culture conditions (37°C, 5% CO_2_) in Smooth muscle Basal Media (SmBM) supplemented with insulin, hFGF-B, gentamicin, amphotericin-B, hEGF, and 5% fetal bovine serum. Supplements such as, human fibroblast growth factor, human epidermal growth factor, insulin, and amphotericin B were added. Cells were subcultured 1 : 5 in 75 cm^2^ culture flasks or in appropriate plates. Cells between passage 7 and 15 were cultured in 6 or 24-well plates and used at 70–90% confluence. Approximately 1 × 10^6^ cells/mL were used in the experiments.

### 2.3. MTT Cytotoxicity Assay

Cells were seeded in 96-well microplate at 3 × 10^5^ cells/mL and then incubated at 37°C in 5% CO_2_. After 24 h, the medium was removed and replaced with fresh medium containing test compounds at various concentrations (twofold dilution) where BA concentration ranged between 0 and 60 *μ*g/mL. Triplicate cultures were established for each treatment. Seventy-two hours later, 20 *μ*L of MTT (5 mg/mL) in PBS solution was added to each well and then the plate was further incubated for 4 h. All remaining supernatant were removed and 150 mL of DMSO was added to each well and mixed thoroughly to dissolve the formed crystal formazan. After 15 minutes of incubation, the absorbance of each well was read at 570 nm using ELISA plate reader (Biotek EL340, USA) [22].

### 2.4. Alkaline Comet Assay

Comet assay was carried out as described previously [23]. Seeded cells in six-well plate were treated with test compound at IC_10_ and IC_25_ for 4 h, and IC_25_ concentration for 24 h for DNA repair studies. Following incubation, detached cells in the medium were collected and added back to trypsinised cells. Then, the suspension was transferred to tube for centrifugation (2500 rpm/5 min at 4°C). The supernatant was removed and pellet was washed with Ca^2+^- and Mg^2+^-free PBS and recentrifuged. The pellets left at the bottom were mixed thoroughly with 80 mL of 0.6% low melting agarose (LMA) (w/v). The mixture was then pipetted onto the hardened 0.6% normal melting agarose (NMA) (w/v) as the first layer gel on the slide. Cover slips were placed to spread the mixture and slides were left on ice for LMA to solidify. Following the removal of the cover slips, the embedded cells were lysed in a lysing buffer containing 2.5 M NaCl, 100 mM Na_2_EDTA, 10 mM Tris, and 1% Triton X-100 for 1 h at 4°C. Slides were soaked in electrophoresis buffer solution for 20 min for DNA unwinding before electrophoresis at 300 mA, 25 V for 20 min. Subsequently, the slides were rinsed with neutralising buffer for 5 min and stained with 50 *μ*L ethidium bromide solution. Slides were left overnight in 4°C before analyzing with fluorescent microscope equipped with 590 nm filter (Leica, Germany). DNA damage scoring was performed on 100 cells where scores between 0 to 4 were given to represent the length of comet tail (0-undamaged, 1-mild damage, 2-moderate damage, 3-severe damage, 4-total damage) according to Heaton et al. [23] and confirmed using KOMET 4.0 analysis package (Kinetic Imaging, Liverpool, UK).

### 2.5. Cell Cycle Analysis

DNA content from VSMCs, treated with betulinic acid with IC_50_ for 24 h, and 48 h was measured by Coulter Epics Altra flow cytometer (Beckman Coulter, USA). Treated and control cells were washed once with 0.01 mol/L PBS (pH 7.2) and fixed in 70% ethanol at 4°C for 18 h. Next, cells were washed once with PBS, and incubated with RNase (50 mg/mL) and propidium iodide (PI, 100 mg/mL) solution for 30 min. The cell cycle phases were detected and apoptosis was quantified by determining the percentage of PI-stained nuclei in the sub-G_1_ peak.

### 2.6. BrdU Cell Proliferation Assay

Proliferation effect of BA-treated VSMCs was studied using BrdU Cell Proliferation Kit (Chemicon, USA). Briefly, VSMCs cells were treated with IC_25_, IC_50_ and IC_75_ of BA for 24, 48 and 72 h. Rapamycin was used as positive control. Two hours before the end of corresponding period, BrdU reagent (20 *μ*L) was added into all well except unstained control 2 h before the end of each treatment time point and further incubated for 16 h. Then, all cells were pelleted, fixed, washed, and labeled with BrdU detection antibody. Then, the unbound antibody was washed and cells were added with goat anti-mouse Ig G, peroxidase-labeled conjugate. After that, 3,3′,5,5′′-tetramethylbenzidine (TMB) substrate was converted by peroxidase conjugate and the reaction was stopped by 2.5 N sulfuric acid stop solution before the plate was read at 450 nm wavelength using *μ* Quant ELISA Reader (BioTek Instruments, USA) at Animal Tissue Culture Laboratory, FBBS, UPM. Each sample and control were assayed in triplicate.

### 2.7. Acridine Orange/Propidium Iodide (AO/PI) Staining

VSMCs were seeded in six-well plate and incubated at 37°C in 5% CO_2_. After 24 h, the medium in each well was removed and replaced with the compound dissolved in medium at IC_50_ for 24 h, 48 h, and 72 h. After incubation, treated and control cells were harvested, washed with PBS, incubated with 5 *μ*L of acridine orange (10 *μ*g/mL) and propidium iodide (10 *μ*g/mL) at a ratio of 1 : 1 in 1 mL of cells, and recentrifuged at 1000 rpm/5 min. After centrifugation, the supernatant was removed leaving 50 *μ*L of remaining supernatant with pellet. The pellet was resuspended and 10 *μ*L of cell suspension was pipetted on slide before putting on cover slip. Within 30 min, the slide was analyzed using fluorescent microscope (Leica, Germany). Viable, apoptotic, and necrotic cells were quantified in a population of 200 cells.

### 2.8. Statistical Analysis

Results were expressed as the mean ± standard error of mean (S.E.M). Statistical analysis was performed with Student's *t*-test for all the assays. Graphs were plotted using Graphpad Prism 5 software.

## 3. Results

### 3.1. Effect of BA on Cell Viability

**Figure 1:**
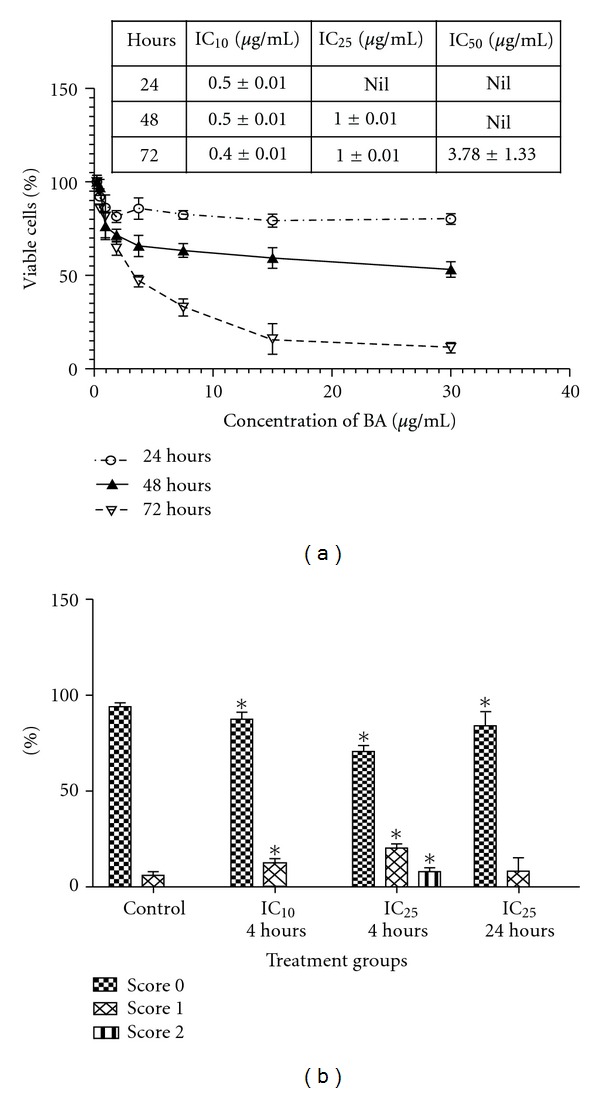
(a) Cell viability after 24 h, 48 h, and 72 h exposure of VSMCs to BA. The data are expressed as a percentage of the control value (value obtained for untreated cells) and the table indicates value of inhibitory concentration at 24 h, 48 h and 72 h. (b) DNA damage scoring following treatment with IC_10_ and IC_25_ of BA for 4 h and 24 h. The results were obtained from three individual experiments. Error bars denote SD *P < 0.05 (Student's-*t* test).

BA showed a concentration-dependent cytotoxic effect on VSMCs at different 24 h, 48 h, and 72 h (Figure 1(a)). The IC_50_ (inhibitory concentration) of BA was about 3.78 *μ*g/mL and this concentration was chosen for further experiments.

### 3.2. Dosage-Dependent DNA Damage Effect of BA

The percentage of DNA damage in VSMCs after treatment with BA for 4 h and 24 h is shown in Figure 1(b). VSMCs treated with BA (IC_10_ = 0.4 *μ*g/mL)-induced DNA damage were observed in 23.4 ± 1.34% cells after 4 h and the damage was at score 1 while BA treatment at IC_25_ (1 *μ*g/mL) showed DNA damage with 22.82 ± 7.83% and 7.5 ± 1.72% of cells were scored 1 and 2, respectively. In negative control group, only a small number of cells showed DNA damage with 94.84 ± 0.59% percentage of cells were scored 0. Treatment of IC_25_ of BA for 24 h showed reduction in cells with DNA damage with score 1 compared to cells treated with IC_25_ of BA for 4 h (14.81 ± 80.97% with score 1 and 8.14 ± 0.98% with score 2). There was no significant increase of VSMCs with DNA damage in a time-dependent manner with BA treatment. There was a statistically significant difference between treated (IC_10_ and IC_25_ BA) and untreated group (negative control) (*P* < 0.05).

### 3.3. BA Arrest VSMC Cell Cycle Progression at G_1_ Phase

The distribution of VSMCs cell cycle phases after BA treatment at IC_50_ = 3.8 *μ*g/mL for 24 h and 48 h is shown in Figure 3. The percentages of G_0_/G_1_ and S phase cells for control group were 59.07 ± 14.82% and 14.63 ± 0.53%, respectively. In contrast, BA (IC_50_ = 3.8 *μ*g/mL) treatment for 24 h led to a significant inhibition of DNA synthesis as evidenced by the fact that the percentages of G_0_/G_1_ phase cells increased to 83.12 ± 6.81% and S phase cells decreased to 4.3 ± 1.61%. The population of G_0_/G_1_ and S phases of VSMCs treated with positive control quiescent for 24 h was 79.55 ± 3.4% and 4.42 ± 0.8%, respectively. Furthermore, the population of G_0_/G_1_ cells and of cells treated with 3.8 *μ*g/mL BA was reduced to 47.52 ± 3.7% after 48 h associated with increase in sub-G_1_ population from 6.55 ± 2.6% at 24 h to 28.58 ± 9.21% at 48 h.

### 3.4. BA Antiproliferative Effect in VSMCs

The effect of BA on VSMCs proliferation was evaluated using BrdU proliferation assay. Antiproliferative effect of BA on VSMCs was dosage- and time-dependent. Untreated cell and cell treated with IC_25_ of BA showed increased of OD from 24 h to 72 h. On the other hand, IC_50_ and IC_75_ of BA treated VSMCs was associated with reduction of OD similar with positive control rapamycin (IC_50_).

### 3.5. BA Induces Apoptosis in VSMCs

Acridine orange and propidium iodide staining methods were used to determine the apoptosis and necrosis rates on VSMCs after 24 h, 48 h, and 72 h incubation with BA. BA significantly increases the number of apoptotic cells and small population of necrotic cells at 48 h and 72 h treatment cells in a dose-dependent manner (Figure 4). The percentage of apoptotic cells at IC_50_ treatment for 24 h were 15.11 ± 1.55% and percentage of apoptotic and necrotic at 48 h period were 23.17 ± 1.73% and 14.63 ± 1.45%, respectively. Further increase of percentage of apoptotic (45.92 ± 1.45%) and necrotic (18.8 ± 1.73%) cells was observed at 72 h.

## 4. Discussion

VSMC is stimulated during progression of restenosis after angioplasty. Thus, natural product such as goniothalamin that possess antiproliferative effect on VSMCs has been suggested as potential antirestenotic agent [7]. In this study, antiproliferative and cytotoxic effects of BA on VSMCs were evaluated using MTT assay, comet assay, flow cytometry cell cycle profiling, BrdU proliferation assay, and AO/PI staining. Based on MTT assay, effect of BA in VSMCs at 24 h incubation was generally not cytotoxic since no IC_50_ was obtained in this study. Comet assay also showed that BA at IC_25_ (1 *μ*g/mL) exhibits low genotoxicity after 4 and 24 h where only early DNA damage was detected in VSMCs. Previous report has showed that BA exerted a cytostatic effect on normal cells at 24 h because they are resistant to cytotoxic stimuli [24]. This effect was similar with another report by Salti et al. [25] who have suggested that BA reduced ultraviolet-C-induced DNA breakage while it mediated apoptosis on melanoma cell via non-p53 pathway.

**Figure 2:**
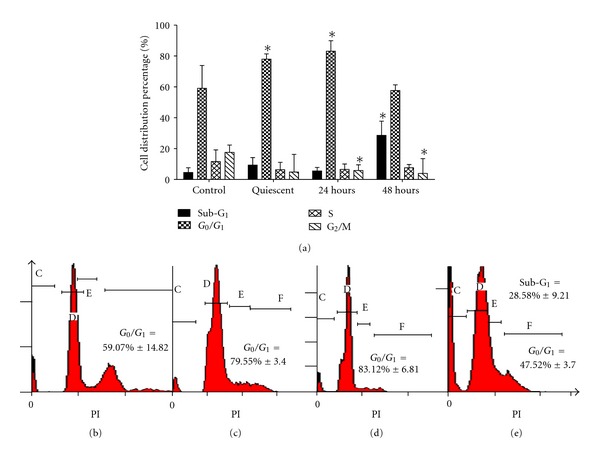
The distribution of VSMCs cell cycle phases after BA treatment at IC_50_ = 3.8 *μ*g/mL for 24 hand 48 h. The results shown are mean ± SD *P* < 0.05 versus control in 3 different experiment (Student's-*t* test). Representative flow cytometry diagram of cell cycle progression for (b) untreated, (c) quiescent treated, (d) IC_50_ BA treated (24 h), and (e) IC_50_ BA treated (48 h) VSMCs.

**Figure 3:**
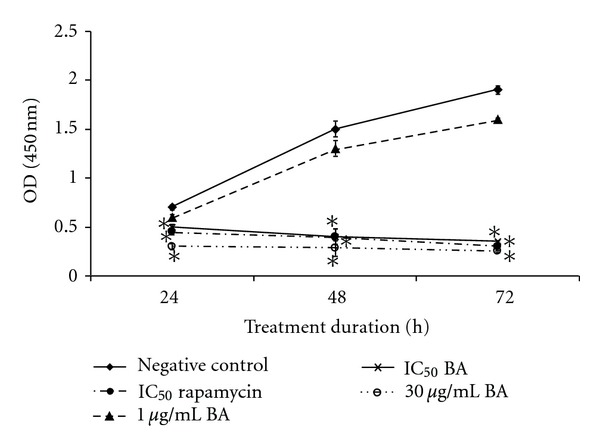
BrdU cell proliferation of VSMCs following treatment with IC_25_, IC_50_, and IC_75_ of BA for 24 h, 48 h and 72 h as compared to untreated and rapamycin-treated cell. The results are the mean ± S.E.M. of three separate experiments. Each data are significantly different among group with *P < 0.05 (Student's-*t* test). Anti-proliferative effects of BA on VSMCs in BrdU proliferation assay. The results shown are mean ± S.D. of OD (570 nm) of control and different treatments for 24, 48, and 72 h.

**Figure 4:**
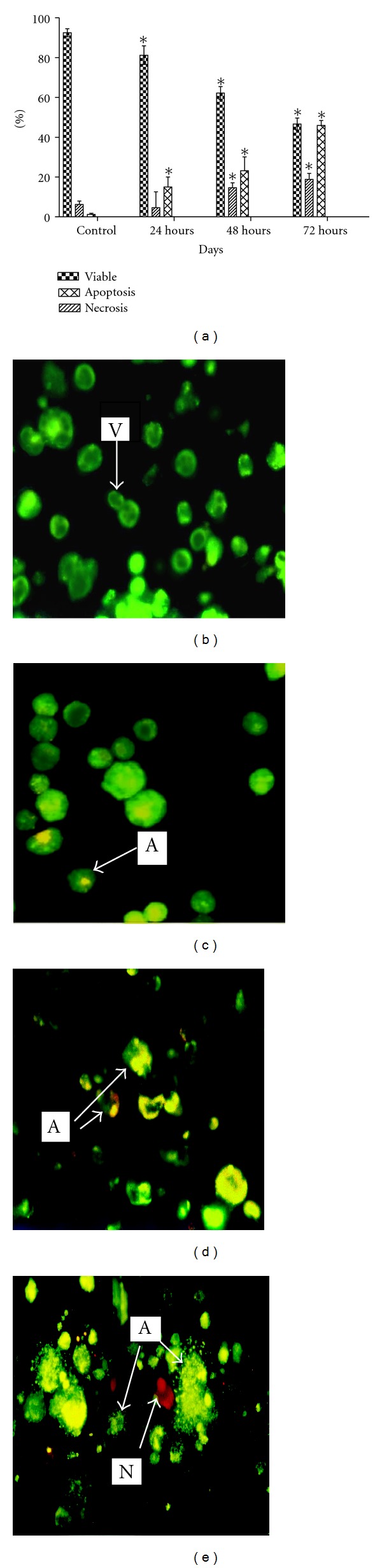
Induction of apoptotic and necrotic cell death by BA in VSMCs after 24 h, 48 h, and 72 h incubation. (a) The number of cells in each of three individual experiments was 100. Error bars denote SD. *P < 0.05 (Student's-*t* test). Representative pictures of acridine orange/propidium for (b) negative control (c) IC_50_ BA for 24 h, (d) IC_50_ BA for 48 h, and (e) IC_50_ BA for72 h. (V: viable, A apoptosis; N: necrosis; SN: secondary necrotic) [400×].

On the other hand, our cell cycle analysis has revealed that IC_50_ (3.8 *μ*g/mL) of BA was able to induce G_1_ phase arrest at 24 h (Figure 2). This result was supported by BrdU proliferation assay where lower OD that indicating lower even of proliferation was detected on IC_50_ and IC_75_ of BA-treated VSMCs similarly with rapamycin (Figure 3). This antiproliferative effect on IC_50_ and IC_75_ of BA-treated VSMCs lasted until 72 h of IC_50_ and IC_75_ of BA treatment. However, treatment of BA at IC_25_ was observed with similar proliferation patent as compared to untreated control. Early G_1_ phase arrest after treatment normally can progress into three possible pathways: remain in G_1_ arrest pending repair, repair the DNA, and return to cell cycle or die via apoptosis [25–27]. Rzeski et al. [28] indicate that BA induced cell cycle arrest at G_1_ phase is likely mediated by the down-regulation of proteins related to the progression of the G_1_ phase. This G_1_ phase arrest has been supported by Yoon et al. [21] who has discovered the antiproliferative effect of VSMCs by BA was through suppression of p21waf1/cip1/p27kip1. The antiproliferation of IC_50_ and IC_75_ of BA treatment can further promote the event of cell death on the VSMCs via apoptosis. This idea was supported by the cell cycle analysis and AO/PI staining at 48 h where DNA fragmentation was observed. Salti et al. [25] have indicated that p53 is not required for eliciting the apoptotic effect by BA. Thus, the apoptotic pathway in proliferation VSMCs with p53 deficient may reflect a shift to p53-independent apoptosis and the growth arrest response of p53 is compromised. Progression of apoptosis may require downstream signaling to induce cell death after 48 h to 72 h. BA has previously shown inhibition of DNA polymerase as well as inhibition of topoisomerases I and II [29]. DNA topoisomerases are ubiquitous enzymes catalyzing changes in the topological state of duplex DNA during, replication transcription, recombination, and DNA repair processes [30]. Thus, interaction between BA and DNA topoisomerase II may be related to induction of early DNA damage and trigger cell cycle arrest at G_1_ phase and followed by induction of apoptosis after 48 h of BA treatment in VSMCs.

In this study, we have provided evidence that VSMCs treated with BA has induced time- and dosage-dependent cell cycle arrest and apoptosis. Data obtained from this work have prospective to further investigate the role of BA in induction of apoptosis but not necrosis in the pathogenesis of VSMCs. Although the molecular responses of BA-induced cell cycle arrest have been confirmed, the molecular responses of BA-induced apoptosis are only partially understood. Thus, future research on the details mechanism of antiproliferative and apoptosis effect of BA on VSMCs needs to be further evaluated. In conclusion, the results obtained have led us to hypothesize that BA induced not only cell cycle arrest but also apoptosis in VSMCs which induced genotoxicity in the dosage-dependent manner. BA can be considered a nontoxic or lack toxic compound that could act as a potent inhibitor to VSMCs.
